# AxonTracer: a novel ImageJ plugin for automated quantification of axon regeneration in spinal cord tissue

**DOI:** 10.1186/s12868-018-0409-0

**Published:** 2018-03-09

**Authors:** Akash Patel, Zhongzhi Li, Philip Canete, Hans Strobl, Jennifer Dulin, Ken Kadoya, Dan Gibbs, Gunnar H. D. Poplawski

**Affiliations:** 1 Department of Neurosciences, 0626, University of California - San Diego, La Jolla, CA 92093 USA; 2 Department of Computer Sciences and Engineering, University of California - San Diego, La Jolla, CA USA; 3 Department of Orthopaedic Surgery, Faculty of Medicine and Graduate School of Medicine, Hokkaido University, Sapporo, Japan; 4 Department of Medicine, National University of Singapore, Singapore, Singapore

**Keywords:** Anatomical tracing, Automated quantification, Axon regeneration, Image analysis, ImageJ, Open-source software, Spinal cord injury, Spinal cord tissue

## Abstract

**Background:**

Quantification of axon regeneration in spinal cord tissue sections is a fundamental step to adequately determine if an applied treatment leads to an anatomical benefit following spinal cord injury. Recent advances have led to the development of therapies that can promote regeneration of thousands of injured axons in vivo. Axon labeling methods and in the application of regeneration-enabling stem cell grafts have increased the number of detectable regenerating axons by orders of magnitudes. Manual axon tracing in such cases is challenging and laborious, and as such there is a great need for automated algorithms that can perform accurate tracing and quantification in axon-dense tissue sections.

**Results:**

We developed “AxonTracer”, a fully automated software algorithm that traces and quantifies regenerating axons in spinal cord tissue sections. AxonTracer is an open source plugin for the freely available image-processing program ImageJ. The plugin identifies transplanted cells grafts or other regions of interest (ROIs) based on immunohistological staining and quantifies regenerating axons within the ROIs. Individual images or groups of images (batch mode) can be analyzed sequentially. In batch mode, a unique algorithm identifies a reference image for normalization, as well as a suitable image for defining detection parameters. An interactive user interface allows for adjustment of parameters defining ROI size, axon detection sensitivity and debris cleanup. Automated quantification of regenerating axons by AxonTracer correlates strongly with semi-manual quantification by the widely-used ImageJ plugin NeuronJ. However, quantification with AxonTracer is automated and reduces the need for user input compared to alternative methods.

**Conclusions:**

AxonTracer is a freely available open-source tool for automated analysis of regenerating axons in the injured nervous system. An interactive user interface provides detection-parameter adjustment, and usage does not require prior image analysis experience. Raw data as well as normalized results are stored in spreadsheet format and axon tracings are superimposed on raw images allowing for subjective visual verification. This software allows for automated, unbiased analysis of hundreds of axon-dense images, thus providing a useful tool in enabling in vivo screens of axon regeneration following spinal cord injury.

**Electronic supplementary material:**

The online version of this article (10.1186/s12868-018-0409-0) contains supplementary material, which is available to authorized users.

## Background

Recently, we reported that grafts of neural progenitor cells (NPCs) or neural stem cells (NSCs) into adult spinal cord lesion sites support robust regeneration of corticospinal tract (CST) axons [1]. To more efficiently screen the effects of therapeutic manipulations on axon regeneration in models of CNS injury, slide scanning microscopes as well as automated analysis software are important. Advances in slide scanning microscopes and data storage capacity make it now possible to generate hundreds of images that contain detailed information of the anatomy of regenerating axons within spinal cord tissue sections. In addition, the development of improved anatomical tracing methods enables more detailed visualization of labeled axons that regenerate after injury. Fully automated, user-friendly software options that allow for rapid quantification of axon regeneration are therefore needed. In particular, in spinal cord injury paradigms where fluorescently labeled cells are grafted readily into injury sites [1–4], it is desirable to analyze axons that are specifically regenerating into cell grafts. The freely available image analysis software ImageJ [5] provides a useful platform for developing new tools to achieve such rapid quantification of newly regenerating axons.

Here, we describe and make freely available an image analysis algorithm we term “AxonTracer” to perform automated analysis of axon growth in spinal cord tissue sections. The algorithm is open-source and based on the free image analysis software program ImageJ [5]. Unlike other non-commercial avenues for axon quantification, the AxonTracer plugin provides a complete, integrated workflow allowing automated detection of fluorescently labeled cell grafts, axon tracing, and quantification with minimal user input. Importantly, AxonTracer produces comparable results to the widely used semi-automated axon quantification plugin NeuronJ [6], but with the benefit of full automation, hence enabling high-throughput image quantification even for very axon-dense samples.

## Implementation

The entirety of the AxonTracer tool is implemented as an ImageJ macro, and can be easily manipulated using a simple text editor. Reference for the ImageJ macro language is available online [7]. AxonTracer can batch process RGB microscope images in various formats (e.g., tiff, .jpeg) and dimensions.

### Data structure

All images that are to be processed together must be in a single folder (analysis folder). The folder cannot contain any other files than image files. The image filenames must be numeric and cannot start with the number “0” (e.g., 01.tiff is not allowed). Images can be of different formats. The numbers do not have to be in consecutive order. For example, a folder containing files with the following names are valid: “3.tiff, 24.jpeg, 25.jpeg, 87.tiff”.

### User interface (UI)

**Fig. 1:**
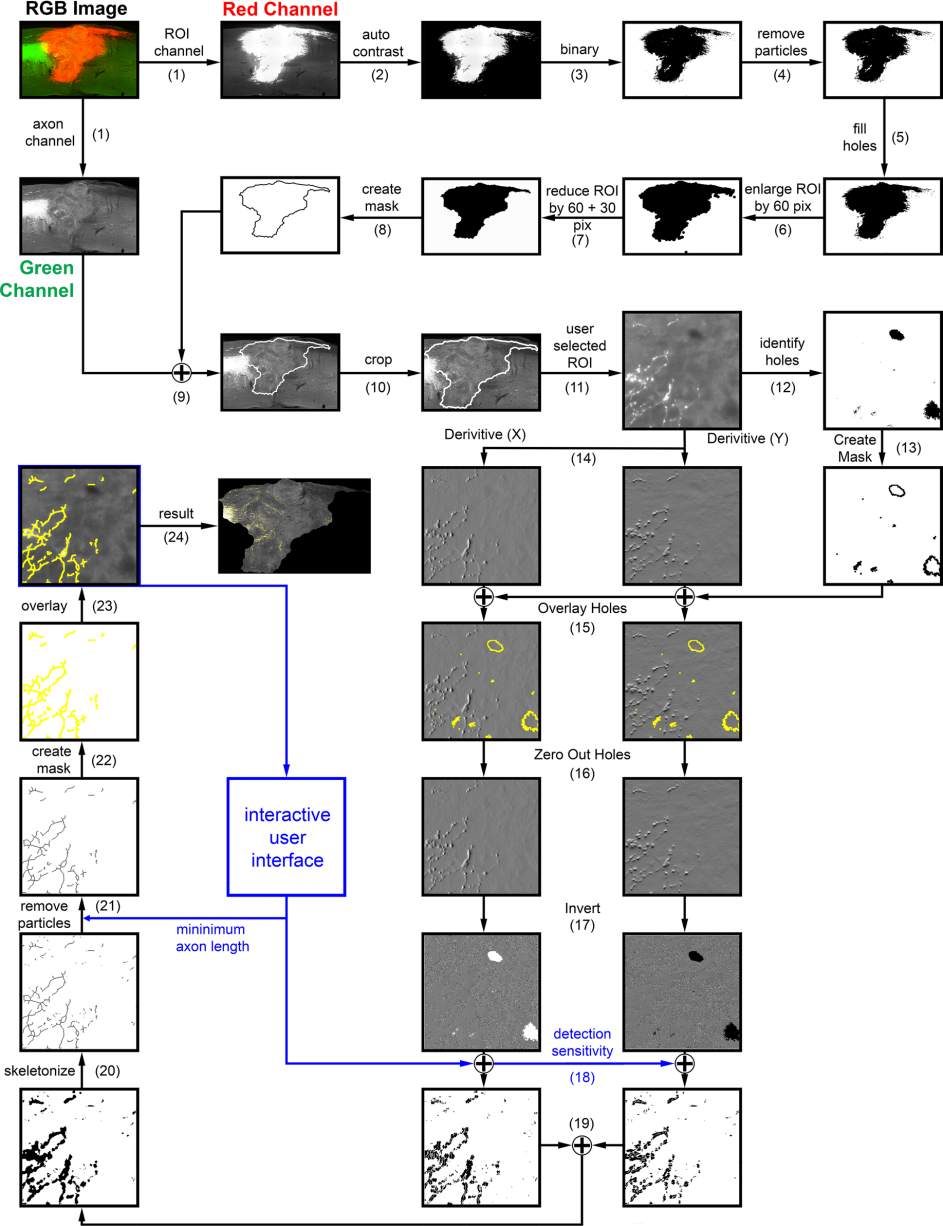
Image Processing Algorithm for Quantification of Axon Regeneration. Flow diagram that illustrates the processing of RGB images fluorescently labeled for CST axons (axon channel) and NPC-graft (ROI channel). Numbers refer to steps in the algorithm description. Blue lines indicate the interactive user interface. (For details refer to main text: Implementation)

**Fig. 2:**
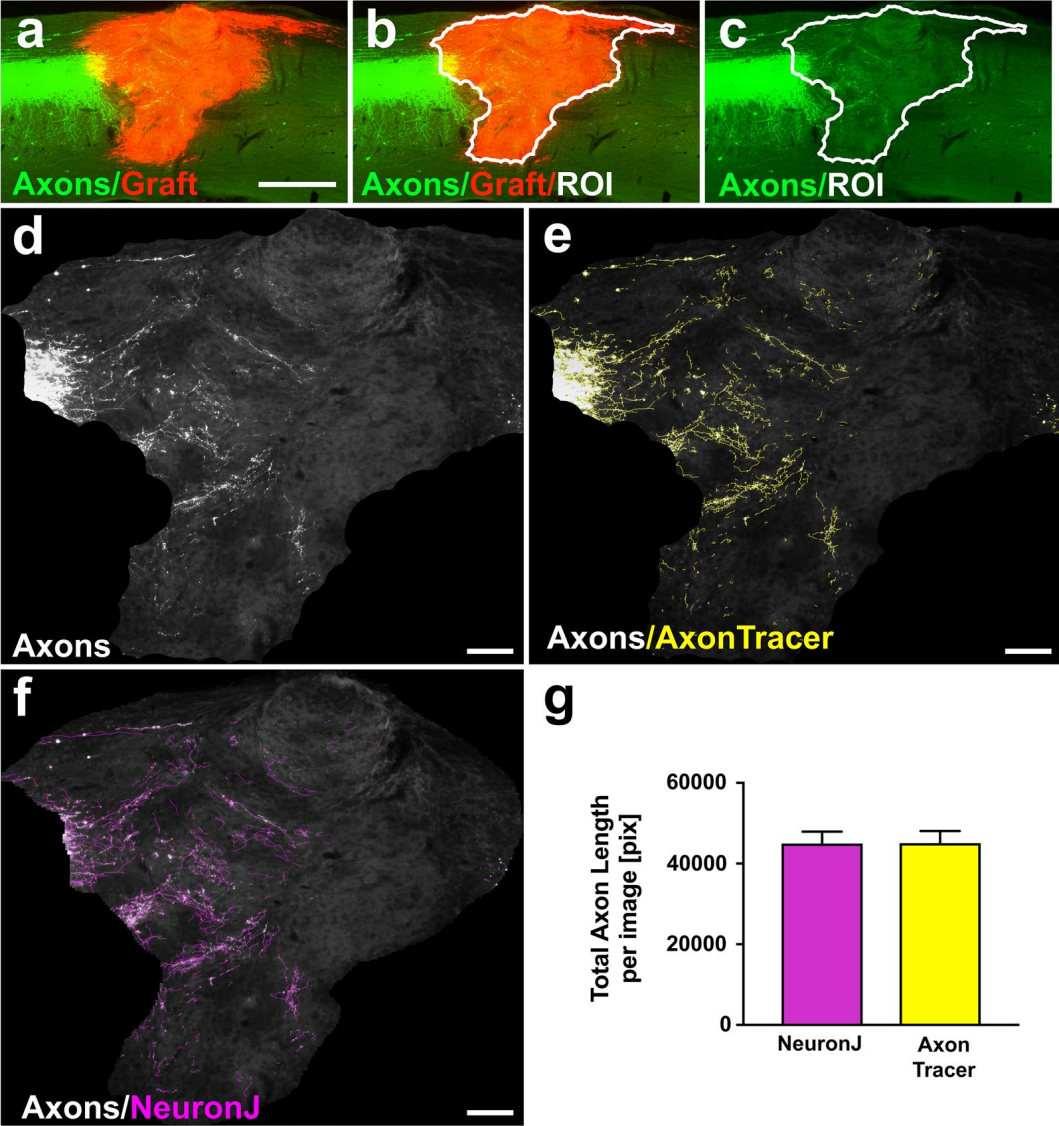
Automatic quantification of regenerating corticospinal axons into fluorescently labeled cell grafts following spinal cord injury. **a** Membrane-targeted sfGFP-AAV8 was injected into the motor cortex of C57Bl/6 mice. 5 days post injection the animals received a dorsal column lesion at cervical level 4 (C4). dsRED-positive spinal cord derived neural precursor cells (Graft) were grafted immediately into the lesion site. 4 weeks later the animals were sacrificed and sagittal sections were stained for sfGFP (Axons) and NPC-graft (Graft). **b** processed image showing automatically detected graft-ROI (white outline). **c** Separate axon channel with graft ROI. **d** Axon channel greyscale image cropped to graft ROI defined in “b”. **e** Automated tracing by AxonTracer (yellow lines) superimposed on axon channel greyscale image shows accurate axon tracing. **f** Semi-automated tracing by NeuronJ superimposed in purple on axon channel greyscale image produces similar tracing results to AxonTracer. **g** Quantification of regenerating axons in graft ROI shows high correlation between AxonTracer and NeuronJ. Mean ± SEM. p = 0.84, *t* test. Scale bar: **a** 500 μm; **d**–**f** 100 μm

**Fig. 3:**
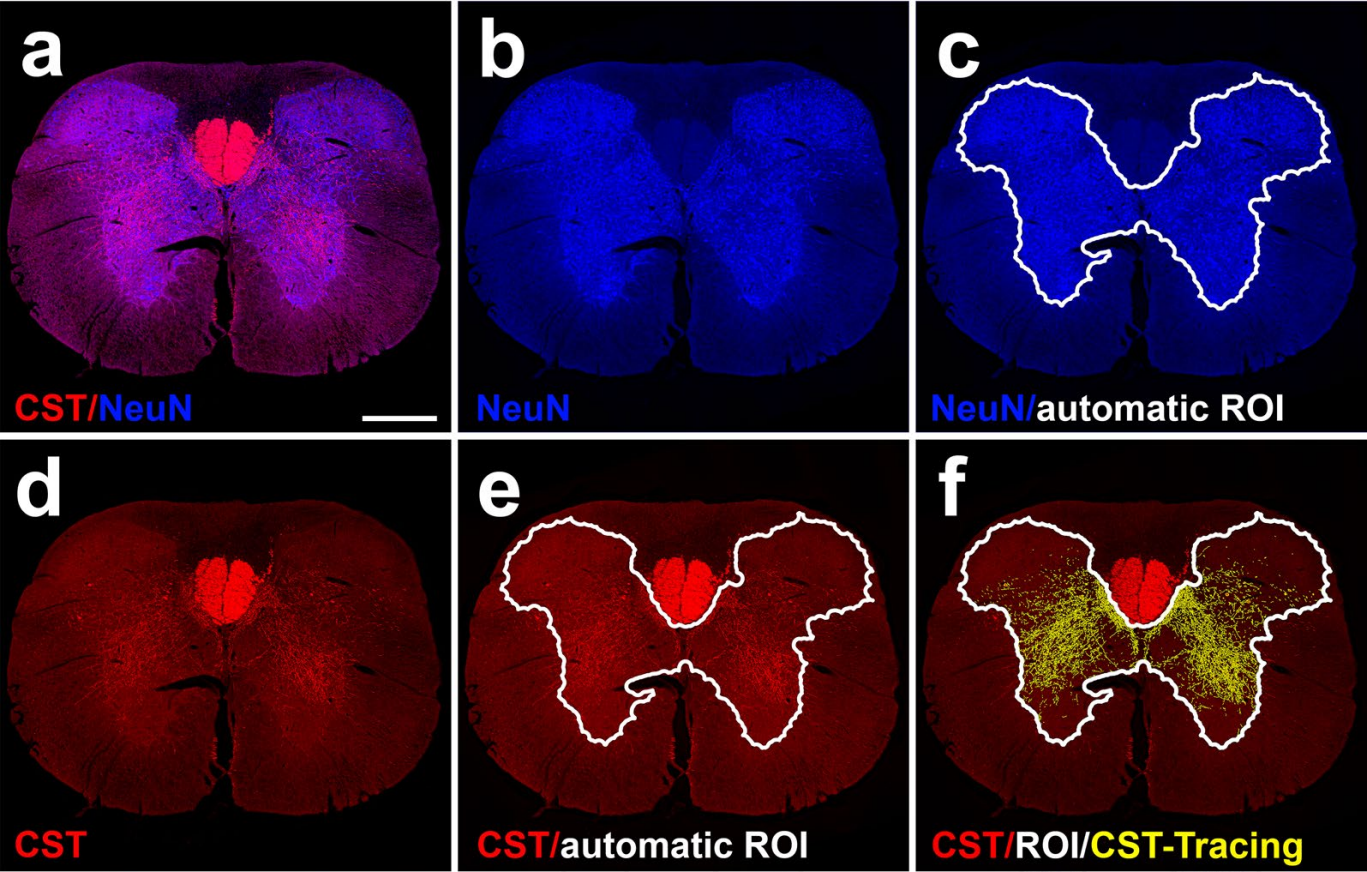
AxonTracer automatically quantifies corticospinal axon sprouting in spinal cord grey matter. **a** Membrane-targeted tdTomato-AAV8 was injected into the motor cortex of adult rats. Transverse spinal cord sections were fluorescently labeled for the cortical spinal tract (CST) and the neuronal cell body marker (NeuN) indicating spinal cord grey matter. **b** ROI channel (NeuN) spilt created by AxonTracer. **c** Automatically detected ROI (white outline) based on NeuN signal outlining spinal cord grey matter. **d** Axon channel (CST) spilt created by AxonTracer. **e** ROI overlay (white outline) on axon channel (CST). **f** Automated tracing (yellow lines) superimposed on axon channel (CST) shows accurate CST axon tracing restricted to spinal cord grey matter. Scale bar: 500 μm

**Fig. 4:**
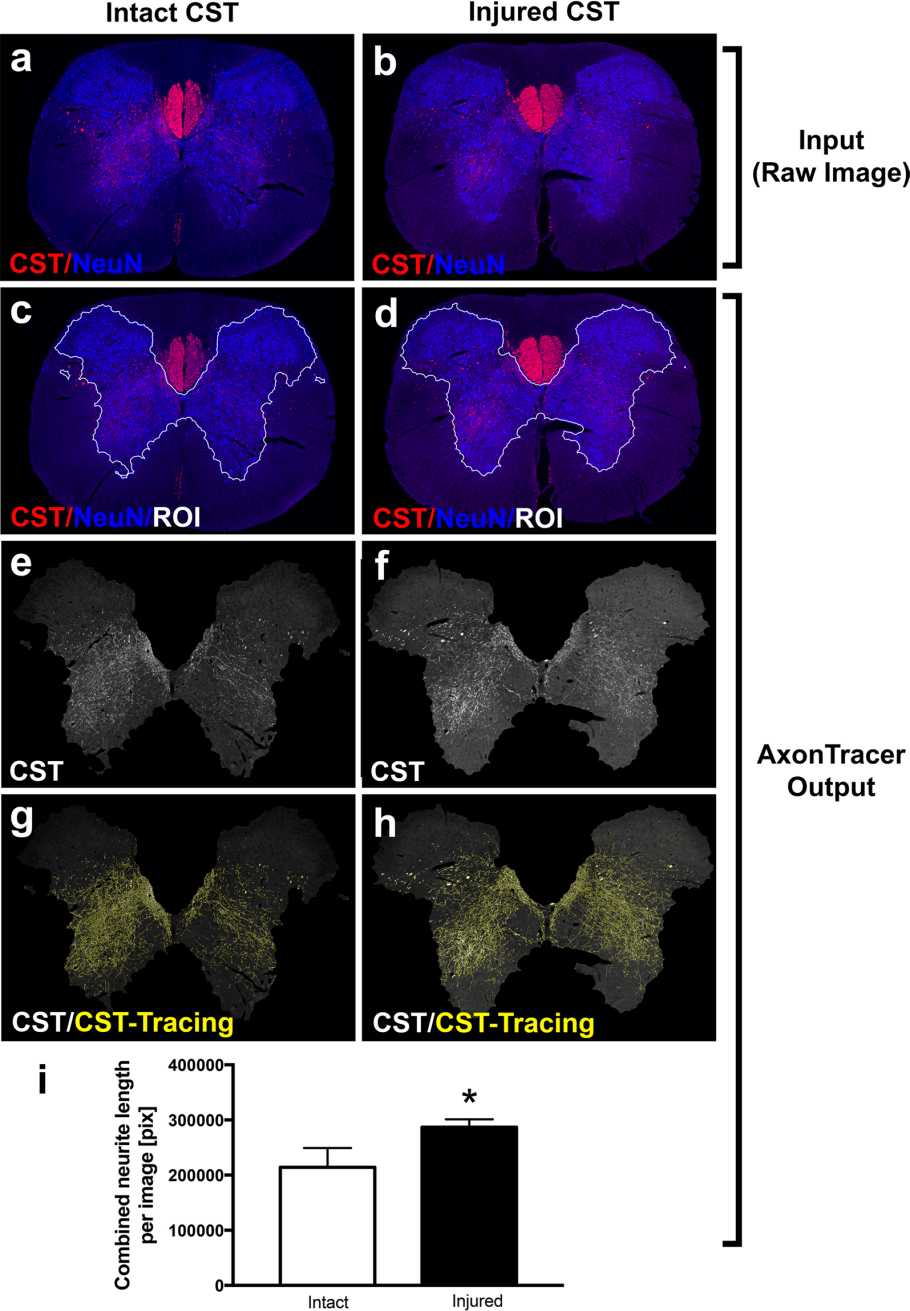
AxonTracer identifies injury induced CST sprouting above level of injury. Membrane-targeted tdTomato-AAV8 was injected into the motor cortex of adult rats. Transverse spinal cord sections at cervical level 2 (C2) were fluorescently labeled for the cortical spinal tract (CST) and the neuronal cell body marker (NeuN) to indicate the spinal cord grey matter. **b, d, f, h** Rats received a cervical (C4) dorsal column lesion 5 days post injection. **a, c, e, g** Rats in uninjured group did not receive a lesion. **a, b** Unprocessed raw images are used for analysis by AxonTracer (Input). **c**–**i** Shows output data automatically created by AxonTracer. **c, d** Input images with ROI (white outline) defined by spinal cord grey matter (NeuN). **e, f** Axon channel in greyscale cropped to ROI showing CST axons in white. **g, h** Automated CST axon tracing superimposed (yellow lines) on greyscale images. **i** Quantification of length of collateral CST axons in grey matter shows increased sprouting in response to injury. Mean ± SEM. p^*^ < 0.05, *t* test

The user initially selects the desired analysis mode. At present, AxonTracer has 4 different analysis modes, but the plugin is easily modifiable to accommodate more modes. All 4 analysis modes use the same algorithm for axon detection, tracing and quantification, and differ only in the degree of automatization regarding the creation of a region of interest (ROI) in which axons are to be detected. These 4 modes are: (1) *Entire Image*: here, no automated or manual ROI for axon detection is created and hence axons in the entire image are analyzed. (2) *Automatic ROI*: here, axon detection is limited to an automatically identified (ROI). The ROI is created based on a fluorescent channel other than the axon channel. In this manuscript, we either use fluorescently labeled cell grafts (Figs. 1, 2), or neuronal cell bodies that are immunohistological stained (Figs. 3, 4) to create the ROI for axon detection. (3) *Manual ROI-1 Axon Channel*: here, axon detection is limited to a user defined ROI. The ROI is actively drawn by the user based on the axon detection channel or a different fluorescent channel if available. (4) *Manual ROI-2 Axon Channels*: here, similar to option 3, axon detection is limited to a user defined ROI. The ROI is actively drawn by the user based on any of the two axon detection channels or a different fluorescent channel. This option allows for quantification of axons in two separate fluorescent channels within the same user defined ROI.

After selecting an analysis mode, the user then assigns the channels (r, g, b) to either axon detection, ROI detection or neither. The user can further decide if the axon tracing data must be normalized (see below). ROI and axon detection parameters are defined and adjusted in an interactive user interface, where ROI size, axon detection sensitivity, and axon cleanup values are set by the user.

### Data normalization

If data normalization is selected, the user will be presented with the image that has the highest fluorescent intensity in the axon channel within the analysis folder. The user is then required to manually draw an ROI around the fluorescent signal that is then used as reference value for all images in the analysis folder. All axon tracing data is saved as raw data (total pixels per image) and as normalized data [raw data/mean intensity of normalization ROI].

### Automatic ROI detection

If automatic ROI detection is selected, the user will be presented with the image within the analysis folder that has the median fluorescent intensity in the ROI channel. An interactive user interface allows for adjustment of the automatically detected ROI by increasing or decreasing the detected area by − 255 to 255 pixels.

### AxonTracer algorithm workflow

Figure 1 shows a detailed overview of the AxonTracer algorithm. First, RGB-images are split into red, green and blue channels (step 1). The channel for ROI detection (R, Fig. 1) is processed separately from the channel selected for axon detection (G, Fig. 1). The ROI channel is processed first. Auto contrast settings are applied to reduce background and increase ROI intensity (step 2), then the image is binarized resulting in a black and white image (step 3). Differences in fluorescent intensity in the ROI channel (e.g. RFP expression in grafted cells) increase the difficulty to automatically define homogenous, continuous ROIs that span the complete graft area. The purpose of steps 4 to 7 is hence to combine smaller fragmented ROIs into larger continuous ROIs and also to eliminate artifacts. First the particle removal filter is applied to subtract small debris from regions outside the ROI (step 4), then the hole filler function is applied to remove the holes inside the ROI to create a more continuous ROI (step 5). The ROIs are then enlarged by 60 pixels (step 6), which leads to the elimination up to 120 pixels wide gaps between adjacent ROIs. The newly formed ROIs are then again reduced first by 60 pixels to counteract then overall increase in size from step 6 and then further reduced in size by 30 pixels (combined reduction of 90 pixels, step 7). This further reduction removes ROIs below the size of 30 pixels that could not be connected to larger ROIs in step 6. We tested pixel ranges from 1 to 500 with diverse image sizes and resolutions to define the ideal parameters for ROI enlargement and reduction. We selected 60 and 90 pixels respectively because they lead to the most consistent results in terms of ROI to graft area representation. These parameters can be adjusted if necessary within the journal itself. They are defined in the beginning of the journal as variables “enlargeROI” and “reduceROI” respectively.

From the remaining black and white image a mask is automatically created using the built-in ImageJ function: “Create Selection” (step 8). This mask is then applied to the axon channel image (green channel, step 9) and the image is cropped to fit the mask (step 10), which reduces the image size and hence improves processing speed. Steps 12–23 are examples of individual axon detection steps that are used when the detection parameters are defined by the user in an interactive process. The same steps are applied to the whole image, once detection parameters are set. First the user selects an area of the axon channel image that will be used in subsequent steps to adjust the axon detection parameters (step 11). The selected area should contain axons that represent the morphology of the entire image, thus detection parameters can be defined adequately. Holes present in the tissue can easily lead to false axon detection along the edges of the holes, since the signal difference between the black background within the hole and tissue around the hole is high and can hence trick the algorithm in detecting artificial axons. To avoid this issue, we identify the holes and fill them average image signal intensity, which reduces false detection of axons. First holes in the tissue are identified (step 12) and a mask is automatically created using two built-in ImageJ function: “Convert to Mask” and then “Create Selection” (step 13). The selection is then saved in the ROI manager and later applied to the derivative images in step 15 to fill the holes with the mean intensity of the image. The original image is processed separately in x- and y-dimensions for steps 14–18. First, the essential differential-geometric image for the x- and y-dimension is computed (step 14), utilizing multi-dimensional, Gaussian-scaled derivatives (https://imagescience.org/meijering/software/featurej/derivatives/). ROIs reflecting holes from step 12 are applied to the images (step 15) and filled with the mean intensity signal of the image (step 16). This step eliminates holes in the tissue that were detected in step 12. Then, the image is inverted pixel by pixel in x- and y-dimension using nested FOR-loops (step 17).

User interface for detection parameter selection: The Parameters for axon detection defined by the user are During the initialization process when the user is adjusting the axon detection parameters step 18 till 23 are repeated with the axon image selected in step 11 until the user accepts the axon detection parameters. Then all images are analyzed with the accepted parameters. And result images are created (step 24) that are then saved in the “traced images” folder.

User defined axon detection parameters are applied to the individual images (step 18). Then both X and Y images are recombined by overlaying both coordinates within the ROI manager (step 19). A well-defined, one pixel wide representation of the fibrous structure is obtained by the essentially topological skeleton function of ImageJ (step 20). The “Analyze particle” function is applied detecting only particles above the user defined cleanup value, thereby eliminating all particles smaller than a user defined pixel size (step 21) and automatically creating a mask of the remaining detected particles. This mask of the remaining skeletons is added to the ROI manager using the built-in ImageJ function: “Create Selection” (step 22) and superimposed with yellow lines (step 23) onto the original image, creating the resulting image (step 24) that is then saved in the “traced images” folder. Once the axon detection parameters are accepted by the user, the algorithm processes all images in the analysis folder with the same settings.

### AxonTracer output

After the analysis is done, the analysis folder contains the original images; the “traced images” folder, depending on the analysis mode selected, contains either all, or some of the following identified by letters “a” to “e” after the filename (e.g., 1a.tiff): (a) the original RGB image with superimposed ROI in white, (b) a cropped grayscale image of axon channel 1 and (c) a cropped grey-scale image of axon channel 1 with superimposed traces in yellow, (d) a cropped grey-scale image of axon channel 2 and (e) a cropped grey-scale image of axon channel 2 with superimposed traces in yellow. The analysis folder will also contain an Excel spreadsheet with quantification results, named “tracing data summary”.

### Identification of individual axons

AxonTracer quantifies the sum of all axons within the entire image or within a specific ROI and traces all axons with the same color (yellow). If quantification of individually traced axons is required, we made an alternate version of the journal, named “AxonTracerDetail”, available for download at www.poplawski-lab.com. “AxonTracerDetail” quantifies the length of each individual axon and creates a multicolor tracing image, where every individual axon is labeled in a unique color. For each image, an individual result datasheet, named “Tracing Data Details.xls” and an image containing color coded axon tracings identifying individual axons, named “Tracing Data Details.png” are created in the folder “Traced Images Detailed Results” (Additional file 1: Fig. S1).

### Critical issues

The unique architecture of AxonTracer allows for accurate axon detection of images with a wide range of signal to noise ratios. However, for images with a high dynamic range that contain both dense axons with high fluorescent signal intensity as well as sparse axons with low fluorescent signal, detection accuracy is reduced. In such cases, AxonTracer yields accurate detection of axons with high fluorescent signal intensity, but a loss of detection of axons with low fluorescent signal intensity, or vice versa. This issue can be greatly improved if images are taken with cameras in HDR (high dynamic range) mode, in which case high fluorescent structures are imaged at lower intensity and vice versa, creating an image with even fluorescent signal distribution.

### Ongoing development

AxonTracer is implemented as an ImageJ macro, and therefore easily modifiable by the user. Updates to the AxonTracer tool will be made available on the AxonTracer website (www.poplawski-lab.com). Due to its open-source implementation, the AxonTracer image analysis software can be adapted to individual analysis needs. Importantly, its usage is not limited to the detection of axons in spinal cord tissue, but can be altered to detect any fibrous structure in uniquely defined ROIs in other tissue sources. We are currently working to expand the capabilities of this plugin. Features in development include automatic segmentation of ROIs, allowing for the quantification of distance of axon regeneration into cell grafts, which is an important measure in the development of functional relays across sites of spinal cord injury [1, 4]. Suggestions to improve the software can be submitted at www.poplawski-lab.com/axontracer.

## Results and discussion

### Automatic quantification of regenerating corticospinal axons growing into fluorescently labeled cell grafts following spinal cord injury

Membrane targeted AAV8-sfGFP (superfolder-GFP) was injected into the motor cortex of C57Bl/6 mice (Jackson Laboratories; n = 4). 5 days post injection, animals received a spinal cord dorsal column lesion at cervical level 4 (C4). dsRED-positive spinal cord derived neural progenitor cells (Graft) were grafted immediately into the lesion site (Fig. 2a). 4 weeks later, animals were sacrificed and sagittal spinal cord sections were stained for sfGFP (Axons) and NPC-graft (Graft) (Fig. 2a). To limit the axon quantification to regenerating axons penetrating the NPC graft, AxonTracer utilizes the red channel to automatically outline the cell graft (Fig. 2b). The Graft outline (white) defines the ROI for axon detection in the green channel (Fig. 2c–e). AxonTracer automatically identifies axons and traces them in yellow, superimposed on the original image (Fig. 2e). To assess whether automated tracing and quantification by AxonTracer produces accurate measurements, we compared the results to semi-manual tracing by the ImageJ plugin NeuronJ [6] (Fig. 2f). Quantification of regenerating axons with both methods was applied to the same images and showed no significant difference (n = 4, p = 0.84 by paired *t* test; Fig. 2g). Additional example images traced with both AxonTracer and NeuronJ are shown in a side-by-side comparison in the supplemental material (Additional file 2: Fig. S2).

The benefit of AxonTracer over other available axon detection analysis algorithms, such as NeuronJ [6], is its fully automated axon tracing, and normalization algorithm. The user does not need any prior image analysis experience, due to AxonTracer’s self-explanatory, interactive user interface, guiding the user step-by-step through the process. When detection parameters are being defined, the software provides a visual confirmation of the detected axons. A second benefit is that analysis by AxonTracer is completely unbiased. Once detection parameters are defined, they are applied to all images in the analysis folder, resulting in identical quantification among experimental groups.

### AxonTracer for automatic quantification of corticospinal axon sprouting in spinal cord grey matter

To further assess the functionality of AxonTracer in spinal cord tissue sections without fluorescently-labeled cell grafts, we next investigated CST axon sprouting in transverse spinal cord tissue sections above level of injury. Delivery of growth-promoting genes has been shown to modulate axonal sprouting within the spinal cord grey matter [8, 9]. To use AxonTracer for automatic quantification of corticospinal tract (CST) axons in transverse spinal cord tissue sections (Fig. 3), we analyzed samples from animals with CST axons traced with membrane-targeted AAV8-tdTomato, and used the neuronal cell body marker NeuN to generate automatic ROIs within the spinal cord grey matter (Fig. 3a–c). Utilizing NeuN labeling for ROI detection excludes the CST main tract label from the analysis and hence limits the analysis to CST axons collaterals with the grey matter (Fig. 3e, f). AxonTracer’s normalization functionality is especially useful in transverse sections, since all virally traced CST axons are present in the main tract of each transverse tissue section. Thus, AxonTracer allows for rapid, unbiased in vivo screening of axon sprouting into spinal cord grey matter (Fig. 3f). To verify the potential of AxonTracer we then investigated injury induced sprouting of the lesioned corticospinal tract in rats [10, 11].

### AxonTracer identifies injury-induced CST sprouting above the level of injury

Lesion of the CST main tract induces sprouting of CST collaterals within the grey matter at spinal cord levels rostral to the injury site [10, 11]. To validate the efficacy of the algorithm, membrane targeted tdTomato-AAV8 was injected bilaterally into the motor cortex of adult Fischer 344 rats (Charles River; n = 9). Half the cohort received a C4 dorsal column lesion 5 days post injection (n = 5). 4 weeks later lesioned animals (n = 5) as well as intact control animals (n = 4) were sacrificed and transverse sections at cervical level 2 (C2) were stained for tdTomato (CST) and spinal cord grey matter (NeuN) (Fig. 4a, b). 3 sections from each animal were analyzed with AxonTracer. Representative, unmanipulated input images (Fig. 4a, b) as well as representative, processed output images created by AxonTracer (Fig. 4c–h) are shown for both groups. Quantification of CST axons in the grey matter shows a significant 37% increase in total axon length in the injured compared to uninjured group (n = 3 sections per animal, *p < 0.05, *t* test; Fig. 4i). The Output images are automatically saved in the folder “traced images” and the axon tracing data is exported in excel format.

## Conclusions

AxonTracer is a freely available open-source tool for automated, unbiased analysis of regenerating axons following spinal cord injury, based on the freely available open-source image analysis platform ImageJ [5]. We demonstrate the application of AxonTracer in two widely used paradigms in spinal cord regeneration research: (1) quantification of injured, regenerating axons into cell grafts and (2) quantification of collateral axon sprouting into spinal cord grey matter. AxonTracer automatically creates publication quality image tracings. Axon quantification data is comparable to semi-manual quantification with the widely used ImageJ plugin NeuronJ [6], with the added benefit of complete automation. An easy-to-use, interactive user interface facilitates all necessary steps from analysis mode selection over detection parameter refinement to data normalization, allowing unexperienced users to exploit AxonTracer’s full capabilities. AxonTracer can easily be adjusted for additional applications for image based quantification of fibrous structures and is such of interest to the broader scientific community.

## Additional files

**Additional file 1: Fig. S1**. AxonTracerDetail quantifies axon length of individual axons and displays multicolor tracings. Shown are fluorescently labeled axons (white) that have either been analyzed with AxonTracer (yellow tracing) or AxonTracerDetail (multicolor tracing). AxonTracer does not differentiate between single axons and quantifies total axon length of all traced axons per image in pixel. AxonTracerDetail utilizes the same axon detection algorithm as AxonTracer but measure axon length of each individually detected fibrous structure. Each individually detected structure has a slightly different color.

**Additional file 1: Fig. S2**. Side-by-side comparison of CST axons in NSC grafts traced with semi-automated NeuronJ or fully automated AxonTracer. Shown are fluorescently labeled axons (white) that have either been analyzed with NeuronJ (purple tracing) or with AxonTracer (yellow tracing) superimposed on axon channel greyscale image. Shown are total sum of traced pixels per image.

## Abbreviations

CST: corticospinal tract
NPC: neural progenitor cell
NSC: neural stem cell
sfGFP: superfolder green fluorescent protein
