# Supplementary figures and images for: AxonTracer: a novel ImageJ plugin for automated quantification of axon regeneration in spinal cord tissue
Source: BMC Neurosci. 2018 Mar 9;19:8. doi: 10.1186/s12868-018-0409-0 (PMC5845359; doi:10.1186/s12868-018-0409-0)

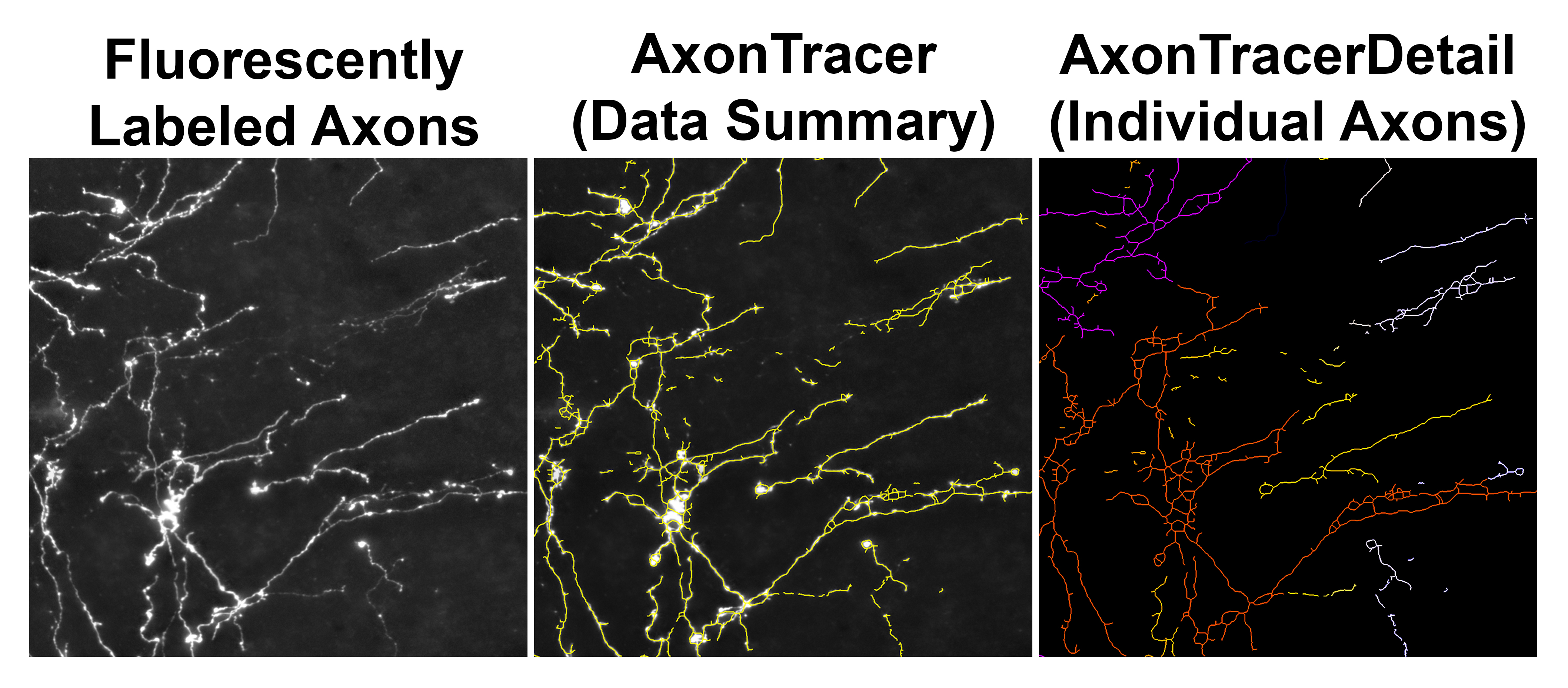

Supplement: Supplementary file 1 — Additional file 1: Fig. S1. AxonTracerDetail quantifies axon length of individual axons and displays multicolor tracings. Shown are fluorescently labeled axons (white) that have either been analyzed with AxonTracer (yellow tracing) or AxonTracerDetail (multicolor tracing). AxonTracer does not differentiate between single axons and quantifies total axon length of all traced axons per image in pixel. AxonTracerDetail utilizes the same axon detection algorithm as AxonTracer but measure axon length of each individually detected fibrous structure. Each individually detected structure has a slightly different color. [file 12868_2018_409_MOESM1_ESM.jpg]

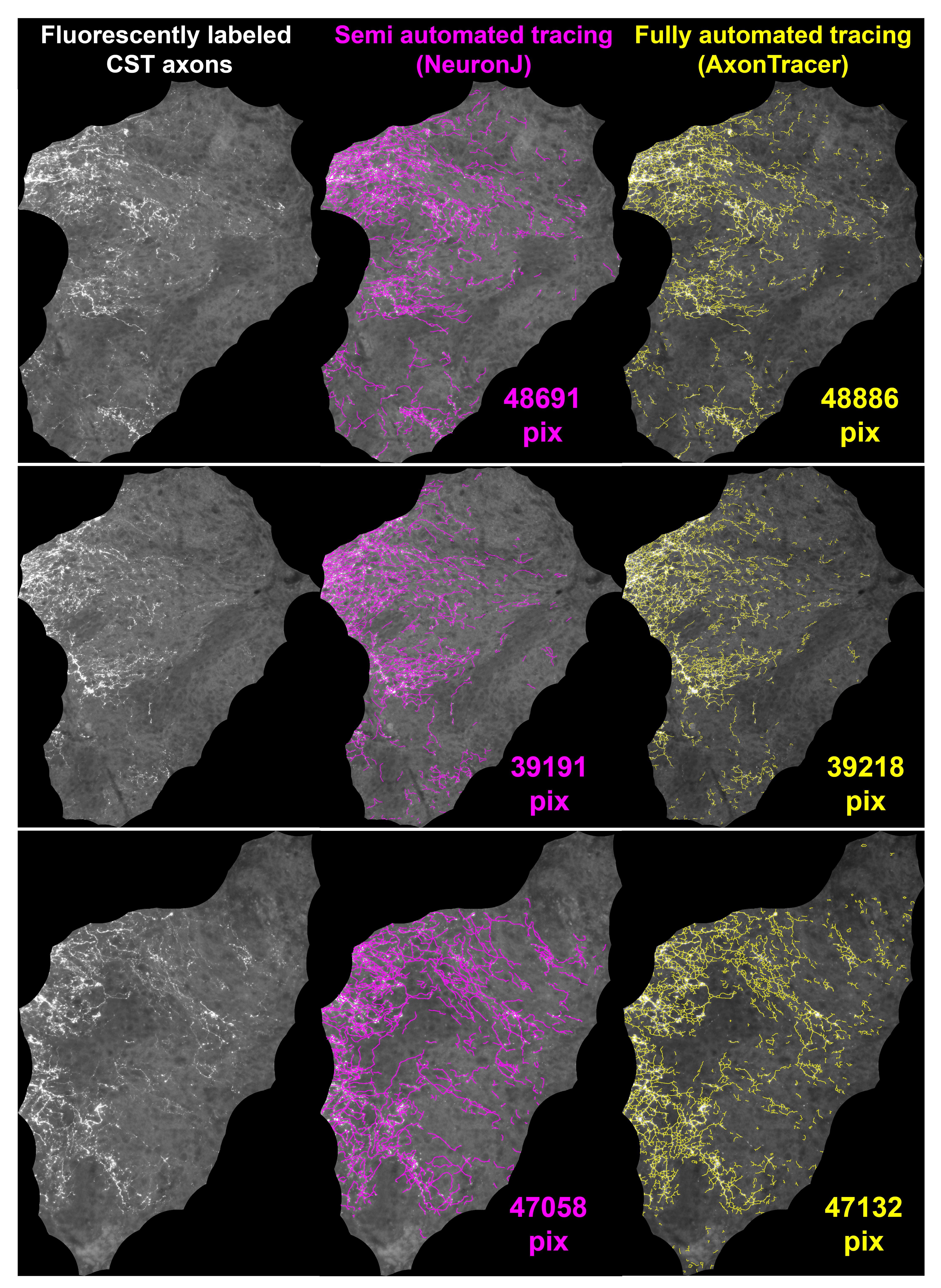

Supplement: Supplementary file 2 — Additional file 1: Fig. S2. Side-by-side comparison of CST axons in NSC grafts traced with semi-automated NeuronJ or fully automated AxonTracer. Shown are fluorescently labeled axons (white) that have either been analyzed with NeuronJ (purple tracing) or with AxonTracer (yellow tracing) superimposed on axon channel greyscale image. Shown are total sum of traced pixels per image. [file 12868_2018_409_MOESM2_ESM.jpg]
